# SARS‐CoV‐2 Infections Among Patients With Liver Disease and Liver Transplantation Who Received COVID‐19 Vaccination

**DOI:** 10.1002/hep4.1853

**Published:** 2021-11-09

**Authors:** Andrew M. Moon, Gwilym J. Webb, Ignacio García‐Juárez, Anand V. Kulkarni, Gupse Adali, David K. Wong, Beth Lusina, George N. Dalekos, Steven Masson, Brandon M. Shore, Eleanor Barnes, A. Sidney Barritt, Thomas Marjot

**Affiliations:** ^1^ Division of Gastroenterology and Hepatology University of North Carolina Chapel Hill NC USA; ^2^ Cambridge Liver Unit Addenbrooke’s Hospital Cambridge University Hospitals Cambridge United Kingdom; ^3^ Department of Gastroenterology Instituto Nacional de Ciencias Médicas y Nutrición Salvador Zubirán Mexico City Mexico; ^4^ Department of Hepatology and Liver Transplantation Asian Institute of Gastroenterology Hospitals Gachibowli Hyderabad India; ^5^ Department of Gastroenterology and Hepatology University of Health Sciences Istanbul Umraniye Training and Research Hospital Istanbul Turkey; ^6^ Toronto Center for Liver Disease Toronto General Hospital Research Institute University Health Network Toronto ON Canada; ^7^ Department of Medicine and Oncology Cumming School of Medicine University of Calgary Calgary AB Canada; ^8^ Department of Medicine and Research Laboratory of Internal Medicine National Expertise Center of Greece in Autoimmune Liver Diseases University Hospital of Larissa Larissa Greece; ^9^ Liver Transplant Unit Freeman Hospital The Newcastle upon Tyne Hospitals NHS Foundation Trust Newcastle upon Tyne United Kingdom; ^10^ Department of Medicine University of North Carolina Chapel Hill NC USA; ^11^ Oxford Liver Unit, Translational Gastroenterology Unit Oxford University Hospitals NHS Foundation Trust University of Oxford Oxford United Kingdom

## Abstract

Many safe and effective severe acute respiratory syndrome coronavirus 2 (SARS‐CoV‐2) vaccinations dramatically reduce risks of coronavirus disease 2019 (COVID‐19) complications and deaths. We aimed to describe cases of SARS‐CoV‐2 infection among patients with chronic liver disease (CLD) and liver transplant (LT) recipients with at least one prior COVID‐19 vaccine dose. The SECURE‐Liver and COVID‐Hep international reporting registries were used to identify laboratory‐confirmed COVID‐19 in CLD and LT patients who received a COVID‐19 vaccination. Of the 342 cases of lab‐confirmed SARS‐CoV‐2 infections in the era after vaccine licensing, 40 patients (21 with CLD and 19 with LT) had at least one prior COVID‐19 vaccination, including 12 who were fully vaccinated (≥2 weeks after second dose). Of the 21 patients with CLD (90% with cirrhosis), 7 (33%) were hospitalized, 1 (5%) was admitted to the intensive care unit (ICU), and 0 died. In the LT cohort (n = 19), there were 6 hospitalizations (32%), including 3 (16%) resulting in mechanical ventilation and 2 (11%) resulting in death. All three cases of severe COVID‐19 occurred in patients who had a single vaccine dose within the last 1‐2 weeks. In contemporary patients with CLD, rates of symptomatic infection, hospitalization, ICU admission, invasive ventilation, and death were numerically higher in unvaccinated individuals. *Conclusion:* This case series demonstrates the potential for COVID‐19 infections among patients with CLD and LT recipients who had received the COVID‐19 vaccination. Vaccination against SARS‐CoV‐2 appears to result in favorable outcomes as attested by the absence of mechanical ventilation, ICU, or death among fully vaccinated patients.

## Graphical abstract

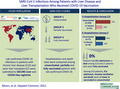

**Abbreviations**
CLD: chronic liver disease
COVID‐19: coronavirus disease 2019
GI: gastrointestinal
ICU: intensive care unit
LT: liver transplant
MMF: mycophenolate mofetil
mRNA: messenger RNA
SARS‐CoV‐2: severe acute respiratory syndrome coronavirus 2

Since the onset of the coronavirus disease 2019 (COVID‐19) pandemic, we have learned much about the sequelae of COVID‐19 in patients with liver disease.^(^ ^1^ ^)^ Patients with decompensated cirrhosis are at increased risk of hospitalization, intensive care unit (ICU) admission, and death from COVID‐19.^(^ ^2^, ^3^, ^4^ ^)^ Although liver transplant (LT) recipients may not be at higher risk of COVID‐19 mortality,^(^ ^5^, ^6^ ^)^ some data suggest that these patients might have an increased risk of severe acute respiratory syndrome coronavirus 2 (SARS‐CoV‐2) infection.^(^ ^7^ ^)^ Meanwhile, vaccine development has progressed at a rapid rate, and vaccine rollouts have tended to prioritize vulnerable patients, including immunocompromised patients.^(^ ^8^ ^)^ While the endorsement and potential value of SARS‐CoV‐2 vaccination is well‐established in patients with liver disease,^(^ ^9^, ^10^, ^11^ ^)^ more data are needed on vaccine response and sequelae of postvaccine infections in patients with chronic liver disease (CLD) and LT recipients.^(^ ^12^ ^)^

There is an excellent immune response to the SARS‐CoV‐2 vaccination in the general population; in vaccinated individuals, complications of COVID‐19, including ICU admission and death, are incredibly rare.^(^ ^13^, ^14^, ^15^, ^16^ ^)^ However, vaccine immunogenicity may not be as robust for immunocompromised or immunosuppressed patients. Patients with advanced liver disease have deficiencies in innate and humoral immunity, referred to as cirrhosis‐associated immune dysfunction.^(^ ^17^, ^18^ ^)^ Similarly, LT recipients require immunosuppressant medications and have blunted antibody responses following influenza, hepatitis A, hepatitis B, and pneumococcal vaccinations.^(^ ^19^, ^20^, ^21^, ^22^ ^)^ Early data suggest that the antibody response to the SARS‐CoV‐2 vaccination is attenuated in CLD^(^ ^23^ ^)^ and solid organ recipients.^(^ ^24^, ^25^ ^)^ However, real‐world data are still lacking on the patterns and outcomes of postvaccination infections in patients with CLD and LT recipients.

We used data from two large international registries to describe cases of laboratory‐confirmed COVID‐19 among patients with CLD and LT recipients who had received vaccination against SARS‐CoV‐2.

## Patients and Methods

We established an online reporting system for cases of laboratory‐confirmed SARS‐CoV‐2 in patients with CLD or prior LT. For this study, data were collected between March 5, 2021, and August 2, 2021 (era after vaccine licensing), through two collaborating registries (SECURE‐Liver supported by the American Association for the Study of Liver Diseases and COVID‐Hep.net supported by the European Association for the Study of the Liver). These registries were coordinated from the University of North Carolina (United States) and the University of Oxford (United Kingdom). Given that all data submitted to these registries were de‐identified, both the University of Oxford Clinical Trials and Research Governance and the University of North Carolina Office of Human Research Ethics deemed that this project was not human research and did not require formal institutional review board approval.

As previously described,^(^ ^2^, ^5^ ^)^ clinicians were invited to submit online case report forms providing de‐identified clinical information on cases of laboratory‐confirmed SARS‐Cov‐2 in patients with prior LT or CLD. Case report forms included demographics, liver disease etiology and severity, SARS‐Cov‐2 vaccination details, and COVID‐19 outcomes, including hospitalization, ICU stay, and death. Clinicians were instructed to submit forms after patients had COVID‐19 for a long enough duration to experience recovery from COVID‐19 or death. Here we describe the cases of SARS‐CoV‐2 infection among patients with at least one prior COVID‐19 vaccine dose compared with contemporary (i.e., submitted March 5, 2021, to August 2, 2021) unvaccinated patients with CLD and prior LT.

## Results

**FIG. 1:**
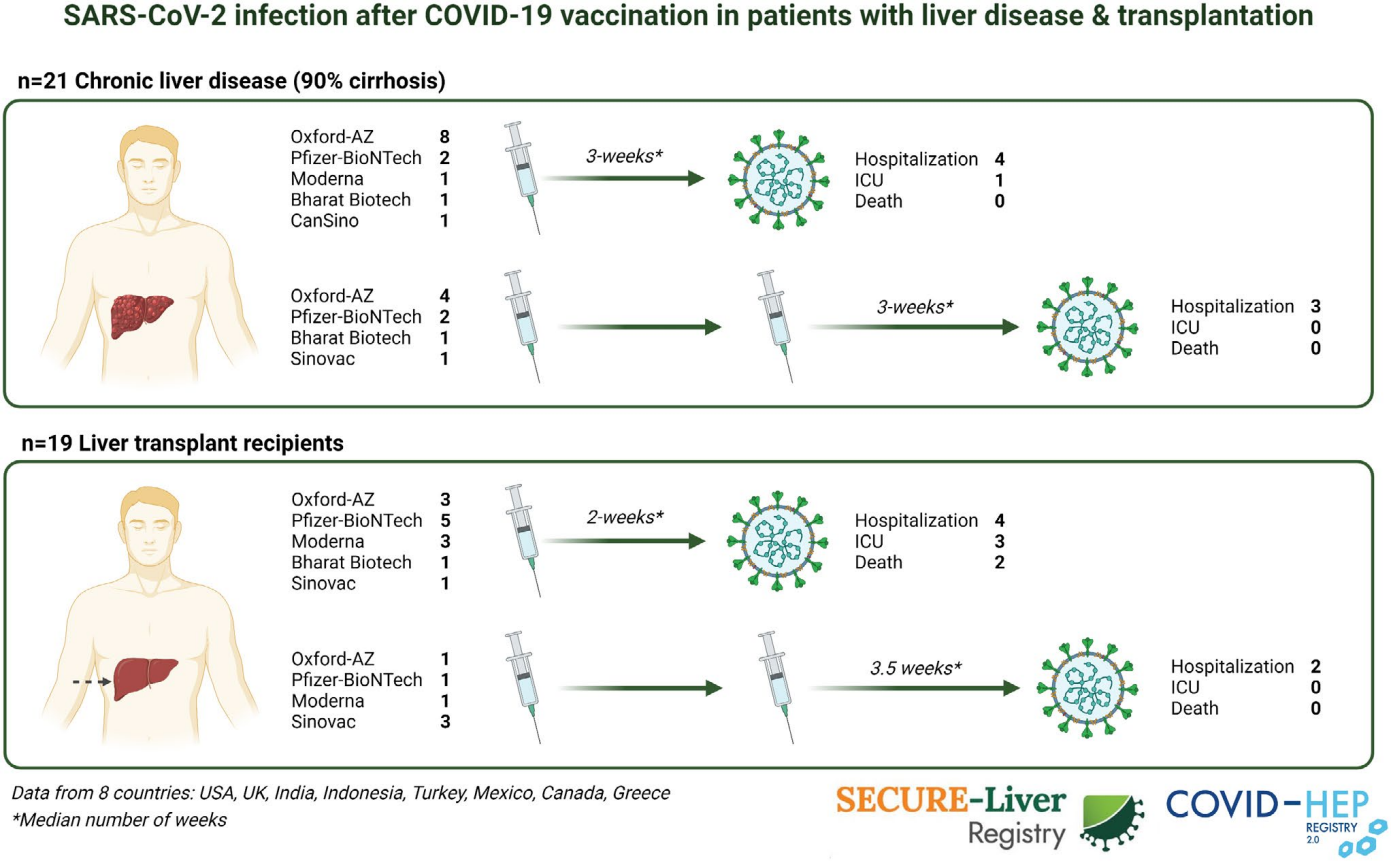
SARS‐CoV‐2 infection after COVID‐19 vaccination in patients with liver disease and transplantation.

A total of 342 cases of lab‐confirmed SARS‐CoV‐2 infection among patients with CLD and prior LT were submitted to SECURE‐Liver and COVID‐Hep 2.0 from March 5, 2021, and August 2, 2021. Among these, 40 (12%) patients (n = 21 with CLD, n = 19 with LT) had at least one prior SARS‐CoV‐2 vaccination, 14 had received both doses (all received the same type of vaccine for both doses), and 12 occurred ≥2 weeks after the second vaccine dose (Fig. 1). Characteristics of CLD and LT cases are found in Tables 1 and 2, respectively, and the description of vaccinations received are shown in Fig. 1.

**TABLE 1:** Patient Characteristics and Information on Vaccination and COVID‐19 Outcomes Among Patients With CLD

Age (years)	Sex	Etiology	CLD Severity	Vaccine Type	Vaccine Doses	Weeks Between v1 & SARS‐CoV‐2	Weeks Between v2 & SARS‐CoV‐2	Hospitalized	Invasive Ventilation	ICU	Death
60‐69	M	HCV	CP‐A	Oxford‐AstraZeneca	1	<1	–	No	–	–	No
50‐59	M	NAFLD	CP‐B	Oxford‐AstraZeneca	1	1	–	No	–	–	No
40‐49	M	NAFLD	CP‐A	Oxford‐AstraZeneca	1	1	–	Yes	No	No	No
40‐49	M	Other	Noncirrhotic CLD	Pfizer‐BioNTech	1	2	–	No	–	–	No
60‐69	M	NAFLD	CP‐A	Oxford‐AstraZeneca	1	2	–	Yes	No	Yes	No
50‐59	F	NAFLD	CP‐A	Oxford‐AstraZeneca	1	3	–	Yes	No	No	No
60‐69	M	HBV	CP‐A	Oxford‐AstraZeneca	1	3	–	No	–	–	No
70‐79	M	Other	Noncirrhotic CLD	Pfizer‐BioNTech	1	3	–	No	–	–	No
60‐69	M	ALD	CP‐A	Moderna	1	4	–	No	–	–	No
50‐59	M	HCV	CP‐A	Bharat Biotech	1	4	–	No	–	–	No
50‐59	F	Other	CP‐A	CanSino	1	4	–	No	–	–	No
40‐49	F	Other	CP‐B	Oxford‐AstraZeneca	1	6	–	No	–	–	No
40‐49	M	ALD	CP‐B	Oxford‐AstraZeneca	1	8	–	Yes	No	No	No
70‐79	F	Other	CP‐B	Sinovac	2	5	1	No	–	–	No
50‐59	F	Other	CP‐C	Bharat Biotech	2	9	1	No	–	–	No
60‐69	M	HCV	CP‐A	Oxford‐AstraZeneca	2	9	2	Yes	No	No	No
60‐69	F	Other	CP‐B	Oxford‐AstraZeneca	2	6	3	No	–	–	No
50‐59	M	NAFLD	CP‐A	Pfizer‐BioNTech	2	9	3	No	–	–	No
70‐79	F	NAFLD	CP‐A	Pfizer‐BioNTech	2	9	6	Yes	No	No	No
60‐69	M	NAFLD	CP‐B	Oxford‐AstraZeneca	2	12	8	Yes	No	No	No
<40	M	Other	CP‐A	Oxford‐AstraZeneca	2	18	6	No	–	–	No

Note:

Full vaccinated patients in gray.

Abbreviations: ALD, alcohol‐associated liver disease; CP‐A, Child‐Pugh A; CP‐B, Child‐Pugh B; CP‐C, Child‐Pugh C; F, female; HBV, hepatitis B virus; HCV, hepatitis C virus; M, male; NAFLD, nonalcoholic fatty liver disease; v1, first vaccine dose; v2, second vaccine dose.

**TABLE 2:** Patient Characteristics Information on Vaccination and COVID‐19 Outcomes Among LT Recipients

Age (years)	Sex	Time Since Transplant (years)	Immunosuppression	Vaccine Type	Vaccine Doses	Weeks Between Last Dose & SARS‐CoV‐2	Weeks Between v2 & SARS‐CoV‐2	Hospitalized	Invasive Ventilation	ICU	Death
50‐59	M	>10	Tacrolimus, MMF	Moderna	1	1	–	Yes	Yes	Yes	Yes
<40	M	5‐10	Tacrolimus	Oxford‐AstraZeneca	1	1	–	No	–	–	No
40‐49	M	1‐5	Tacrolimus	Sinovac	1	1	–	No	–	–	No
40‐59	F	1‐5	Tacrolimus, MMF	Oxford‐AstraZeneca	1	1	–	No	–	–	No
<40	M	5‐10	Tacrolimus	Pfizer‐BioNTech	1	2	–	No	–	–	No
60‐69	M	<1	Sirolimus, MMF	Moderna	1	2	–	Yes	Yes	Yes	Yes
70‐79	F	>10	Sirolimus	Pfizer‐BioNTech	1	2	–	Yes	Yes	Yes	No
60‐69	F	>10	Tacrolimus	Pfizer‐BioNTech	1	2	–	No	–	–	No
60‐69	F	1‐5	Tacrolimus, Everolimus, MMF	Bharat Biotech	1	2	–	No	–	–	No
60‐69	M	1‐5	Tacrolimus	Pfizer‐BioNTech	1	2	–	No	–	–	No
40‐49	F	>10	Prednisone, tacrolimus, MMF	Pfizer‐BioNTech	1	3	–	No	–	–	No
60‐69	M	1‐5	Tacrolimus	Moderna	1	3	–	No	–	–	No
70‐79	M	6‐10	Tacrolimus, azathioprine	Oxford‐AstraZeneca	1	5	–	Yes	No	No	No
<40	M	1‐5	Prednisone, tacrolimus	Moderna	2	6	2	No	–	–	No
60‐69	M	>10	Tacrolimus, MMF	Pfizer‐BioNTech	2	12	2	Yes	No	No	No
60‐69	F	1‐5	Not known	Sinovac	2	7	3	No	–	–	No
50‐59	F	5‐10	Tacrolimus	Sinovac	2	8	4	Yes	No	No	No
60‐69	F	1‐5	Tacrolimus	Sinovac	2	8	4	No	–	–	No
40‐49	M	1‐5	Tacrolimus, MMF	Oxford‐AstraZeneca	2	20	12	No	–	–	No

Note:

Full vaccinated patients in gray.

Abbreviations: F, female; M, male; v1, first vaccine dose; v2, second vaccine dose.

### COVID‐19 in Patients with CLD With ≥1 Vaccine Dose Versus Unvaccinated

In patients with CLD who received at least one vaccination (n = 21), the median age was 59 years (range 28‐72), 67% were male, and cases were from North America (n = 10, 45%), India (n = 6, 27%), the United Kingdom (n = 2, 9%), Turkey (n = 1, 5%), Indonesia (n = 1, 5%), and Greece (n = 1, 5%) (Table 1). The most common CLD etiology reported was nonalcoholic fatty liver disease (n = 7, 33%), and 90% of patients had cirrhosis (Child Pugh A/B/C, 63%/32%/5%). SARS‐CoV‐2 infection was diagnosed after one vaccine dose in 13 (62%) and after two doses in 8 (38%). Viral vector vaccines (Oxford‐AstraZeneca, CanSino) were received by 13 (62%), messenger RNA (mRNA) vaccines (Pfizer‐BioNTech, Moderna) by 5 (24%), and inactivated vaccines (Bharat Biotech, Sinovac) by 3 (14%). Among those who received a single vaccine dose, the median time from vaccination to infection was 3 weeks (range 0‐8), and for those who received both doses, the median time from the second dose to infection was 3 weeks (range 1‐8). Regarding symptoms at presentation, 11 (57%) had respiratory symptoms, 1 (5%) had gastrointestinal (GI) symptoms, 1 (5%) had GI and respiratory symptoms, and 8 (38%) reported no GI or respiratory symptoms (Table 3). Of these 21 patients who received prior vaccination, 7 (33%) were hospitalized, 1 (5%) was admitted to the ICU, 0 required invasive ventilation, and 0 died. Among patients with cirrhosis (n = 19), 9 (47%) developed a new decompensating event (8 new/worsening ascites, 7 hepatic encephalopathy, 4 spontaneous bacterial peritonitis), including 5 patients who were fully vaccinated (≥2 weeks after second dose).

**TABLE 3:** Symptoms, Hospitalization, ICU Admission, Invasive Ventilation, and Death by Liver Disease and Vaccination Status

	All CLDs, ≥1 Vaccine Dose (n = 21)	All CLDs, Unvaccinated (n = 225)	Cirrhosis, ≥1 Vaccine Dose (n = 19)	Cirrhosis, Unvaccinated (n = 159)	Prior LT, ≥1 Vaccine Dose (n = 19)	Prior LT, Unvaccinated (n = 77)
GI and/or respiratory symptoms (n, %)	13 (62%)	172 (76%)	12 (63%)	118 (74%)	17 (89%)	68 (88%)
Hospitalization (n, %)	7 (33%)	162 (72%)	7 (37%)	118 (74%)	6 (32%)	33 (43%)
ICU admission (n, %)	1 (5%)	20 (9%)	1 (5%)	15 (9%)	3 (16%)*	7 (9%)
Invasive ventilation (n, %)	0 (0%)	12 (5%)	0 (0%)	9 (6%)	3 (16%)*	9 (12%)
Death (n, %)	0 (0%)	18 (8%)	0 (0%)	15 (9%)	2 (11%)*	6 (8%)

* All among patients with a single dose within 1‐2 weeks of SARS‐CoV‐2 diagnosis.

In comparison, among the 225 unvaccinated patients with CLD with COVID‐19 (median age 59 years, 62% male, 72% cirrhosis, Child‐Pugh A/B/C 47%/31%/22%), 185 (76%) had respiratory and/or GI symptoms from COVID‐19 compared with 57 (24%) who did not endorse GI or respiratory symptoms. Of these unvaccinated CLD cases, 162 (72%) were hospitalized, 20 (9%) were admitted to the ICU, 12 (5%) required invasive ventilation, and 18 (8%) died, primarily from COVID‐19 lung disease (n = 11 of 18, 61%) (Table 3). Similarly, rates of symptomatic infection, hospitalization, ICU admission, invasive ventilation, and death were numerically higher in unvaccinated compared to vaccinated patients with cirrhosis (Table 3).

### COVID‐19 in LT Recipients With ≥1 Vaccine Dose Versus Unvaccinated

Among LT recipients who received at least one vaccination (n = 19), the median age was 60 years (range 32‐79), 58% were male, and most were from North America (n = 14, 74%) followed by Turkey (n = 2, 11%), India (n = 2, 11%), and the United Kingdom (n = 1, 5%) (Table 2). The median time from LT to infection was 4 years (range 1‐24), and the most common immunosuppression regimen prior to diagnosis was tacrolimus monotherapy (n = 8, 42%). A total of 7 (37%) were on mycophenolate mofetil (MMF). The most frequent vaccine type was mRNA (n = 10, 53%), and the remaining patients received inactivated (n = 5, 26%) and viral vector vaccines (n = 4, 21%). Among those who received a single vaccine dose, the median time from the vaccination to infection was 2 weeks (range 1‐5), and for those who received two doses, the median time from the second vaccine dose to infection was 3.5 weeks (range 2‐12). Most (n = 13, 68%) patients had respiratory symptoms at diagnosis, and 1 (5%) had no respiratory or GI symptoms (Table 3). There were 6 hospitalizations (32%), including 3 (16%) resulting in mechanical ventilation and ICU admission and 2 (11%) resulting in death. Deaths were from COVID‐19 lung disease in 1 patient and cardiogenic shock in 1 patient. Of note, all three cases of severe COVID‐19 occurred in patients who had a single vaccine dose within the last 1‐2 weeks (i.e., not fully vaccinated). Among fully vaccinated LT recipients (n = 5), there were 2 hospitalizations and 0 ICU admissions or deaths.

There were 77 unvaccinated LT recipients with lab‐confirmed COVID‐19 (median age = 53 years, 55% male, median time from transplant = 6 years [range <1‐31 years], 48% on tacrolimus monotherapy/29% on prednisone/22% on MMF). Respiratory symptoms were reported in 29 (38%), GI symptoms in 3 (4%), both GI/respiratory symptoms in 36 (47%), and neither respiratory nor GI symptoms in 8 (10%). Of these unvaccinated LT recipients, 33 (43%) were hospitalized, 7 (9%) were admitted to the ICU, 9 (12%) required mechanical ventilation, and 6 (8%) died, all from COVID‐19 lung disease (Table 3).

## Discussion

In the general population, SARS‐CoV‐2 vaccination has demonstrated excellent safety and efficacy, resulting in a robust immune response and significantly reduced risks of COVID‐19 infection and complications.^(^ ^16^ ^)^ As we have previously shown, patients with decompensated cirrhosis have increased risks of COVID‐19 complications resulting in ICU admission and death.^(^ ^2^ ^)^ Given the potential for higher risks of COVID‐19 in CLD and LT recipients, guidelines strongly recommend vaccination in patients with liver diseases.^(^ ^9^, ^10^, ^11^ ^)^ In patients with CLD and prior LT, there is a lingering concern of low vaccine immunogenicity, given underlying immune dysfunction.^(^ ^12^ ^)^ This case series adds to recently published literature on breakthrough infections in transplant recipients,^(^ ^26^ ^)^ demonstrating the potential for breakthrough SARS‐CoV‐2 infections in patients with CLD and LT recipients. Yet, reassuringly, among patients who were fully vaccinated, there were no cases resulting in ICU admission, invasive ventilation, or death. Furthermore, among patients with CLD and cirrhosis, rates of hospitalization, ICU admission, invasive ventilation, and death were numerically lower among patients who had received at least one vaccine dose compared with those who were unvaccinated. These findings are consistent with recently published papers reporting that SARS‐CoV‐2 vaccination reduces severe disease in patients with cirrhosis^(^ ^27^ ^)^ and solid organ transplant recipients.^(^ ^28^ ^)^

Data on COVID‐19 vaccine response primary consist of reports of postvaccination antibody titers in LT recipients.^(^ ^24^ ^)^ Antibody titers are not the only correlate of protection against SARS‐CoV‐2 infection and may not be a sufficient measure of protection against COVID‐19 in patients with CLD and LT recipients. It may be that antibodies help prevent infections, which could explain breakthrough infections in our series. However, T‐cell responses, which may be preserved and sufficient in patients with CLD and LT recipients, might help attenuate the COVID‐19 disease course. This could underlie the low rates of ICU admission, mechanical ventilation, and death in patients with CLD who received the full COVID‐19 vaccine series in this data set. Supporting these observations, a recent report suggests that heart and liver transplant recipients develop adequate humoral or cellular responses to an mRNA vaccine.^(^ ^29^ ^)^ Postvaccination T‐cell responses in patients with CLD have yet to be fully characterized.

The few cases of severe COVID‐19 resulting in ICU admission and death reported in this series occurred after a single dose of SARS‐CoV‐2 vaccination and were diagnosed within 1‐2 weeks after the vaccine dose. SARS‐CoV‐2 immunity continues to improve beyond 2 weeks of the first mRNA vaccine dose and increases further after a second dose.^(^ ^30^ ^)^ It is also possible that some of these patients had SARS‐CoV‐2 infection at the time of vaccination. These data are important to inform patients on the need to complete the SARS‐CoV‐2 vaccination series and use other recommended strategies to prevent SARS‐CoV‐2 acquisition.

Among fully vaccinated patients, there were no cases of COVID‐19 resulting in ICU admission, mechanical ventilation or death, which should provide reassurance to patients and providers. However, there were some cases in our series of liver‐related decompensation events in fully vaccinated individuals, and the potential for breakthrough cases among an immunosuppressed population raises questions about improved strategies of protection against COVID‐19 in vulnerable populations. There has been interest in providing a booster dose to immunosuppressed patients, who may not mount a robust immune response. In a recent case series of vaccinated transplant recipients, a third of patients with negative antibody titers after two doses had increased titers after a third SARS‐CoV‐2 vaccination dose.^(^ ^31^ ^)^ However, a large proportion of patients had persistently low or negative anti‐spike antibody titers after a third booster dose. Until more data are available on the benefits of this strategy, this suggests that precautions including social distancing and masking should remain in place for patients at the highest risk of severe COVID‐19, including those with decompensated cirrhosis.^(^ ^2^ ^)^

This study is strengthened by its large, international, diverse patient data set. Additionally, the use of clinician reporting minimizes the risk of misclassification of liver disease, vaccination details, and COVID‐19 outcomes. However, the findings of this study have to be interpreted in the context of potential limitations. First, this cohort may be subject to selection bias inherent in registry studies, potentially resulting in an overrepresentation of patients with severe COVID‐19. Second, this study design does not allow for the identification of patients with liver disease who were vaccinated but did not test positive for SARS‐CoV‐2. Therefore, while these data demonstrate the potential for breakthrough infections, we are unable to determine the rates of COVID‐19 infections among vaccinated patients with liver disease. Third, as a comparison population, we included cases of SARS‐CoV‐2 among unvaccinated patients with CLD and liver transplant recipients. While these cases were submitted to registries during an identical time period to vaccinated cases (March 5, 2021, to July 22, 2021), it remains possible that some of the infections among unvaccinated individuals occurred before this time (such as before vaccines were available) and were logged after some delay. Finally, we were not able to adjust for potential center‐specific or region‐specific differences in the management of COVID‐19, which could influence hospitalization rates, ICU escalation decisions, and outcomes.

In summary, this case series demonstrates the potential for breakthrough COVID‐19 infections among patients with CLD and LT recipients. Although some fully vaccinated patients were hospitalized, reassuringly, COVID‐19 resulting in ICU admission or death was limited to patients who had received a recent, single SARS‐CoV‐2 dose. These data reinforce the existing guidelines recommending COVID‐19 vaccination for patients with CLD and LT recipients. However, well‐established strategies for COVID‐19 prevention, including physical distancing and masking, should be considered even for vaccinated patients with CLD and LT recipients. Given the remaining unknowns, ongoing research in these populations on postvaccine immune response and strategies such as booster doses will be important.

## Supporting information

Table S1
