# Supplementary material for: SARS‐CoV‐2 Infections Among Patients With Liver Disease and Liver Transplantation Who Received COVID‐19 Vaccination
Source: Hepatol Commun. 2021 Nov 9;6(4):889–97. doi: 10.1002/hep4.1853 (PMC8652790; doi:10.1002/hep4.1853)
Supplement: Supplementary file 1 — Table S1 [file HEP4-6-889-s001.docx]

**Supplementary Table 1 – Full list of contributing clinicians and patients submitted to the SECURE-Liver and COVID-Hep Registries (5 March 2021-2 August 2021)**

| **Contributor** | **Provider** | **Institution** |
| --- | --- | --- |
| Anand V Kulkarni | Anand V Kulkarni | Asian Institute of Gastroenterology Hospitals, Hyderabad, India |
| Andrew Moon | Jama Darling | University of North Carolina, Chapel Hill, NC, USA |
| Antonietta Romano | Antonietta Romano | Madre Teresa di calcutta Hospital Monselice, Padua, Italy |
| Beth Lusina | Kelly Burak | Southern Alberta Liver Transplant Program, Calgary, Canada |
| Beth Lusina | Meredith Borman | Southern Alberta Liver Transplant Program, Calgary, Canada |
| Brandon Shore |  | University of North Carolina, Chapel Hill, NC, USA |
| Colina Yim | Colina Yim | Toronto Centre for Liver Disease, Toronto, Canada |
| David Wong | David Wong | University of Toronto, Toronto, Canada |
| Deirdre Noone | Professor John Ryan | Liver Support Unit Beaumont Hospital, Dublin, Ireland |
| Dimitris Basoulis |  | Laiko General Hospital, Athens, Greece |
| Akash Raut | Sagar Poudyal | Chitwan Medical College, Bharatpur, Nepal |
| Habib Abdulnabi Abdulla | Jawad Khamis | Salmaniya Medical Complex, Manama, Bahrain |
| Fardah Akil | Fardah Akil | Dr Wahidin Sudirohusodo General Hospital, Makassar City, Indonesia |
| Gualano Gisela | Gisela Gualano | Hospital Regional de Santiago del Estero, Argentina |
| Gupse Adali | Gupse Adali | University of Health Sciences Istanbul Umraniye Training and Research Hospital, Istanbul, Turkey |
| Heidi Drescher | Casey Kolendich | Montana Gastroenterology, Missoula, Montana, USA |
| Ignacio García Juárez | Ignacio García Juárez | Instituto Nacional de Ciencias Medicas y Nutrición Salvador Zubirán, Mexico City, Mexico |
| Isaac Ruiz | Catherine Vincent | Centre Hospitalier de l'Université de Montréal (CHUM), Montreal, Canada |
| Isaac Ruiz | Claire Fournier | Centre Hospitalier de l'Université de Montréal (CHUM), Montreal, Canada |
| Isaac Ruiz | Daphna Fenyves | Centre Hospitalier de l'Université de Montréal (CHUM), Montreal, Canada |
| Isaac Ruiz | Denis Marleau | Centre Hospitalier de l'Université de Montréal (CHUM), Montreal, Canada |
| Isaac Ruiz | Geneviève Huard | Centre Hospitalier de l'Université de Montréal (CHUM), Montreal, Canada |
| Isaac Ruiz | Isaac Ruiz | Centre Hospitalier de l'Université de Montréal (CHUM), Montreal, Canada |
| Magda Meszaros | Magda Meszaros | Montpellier, Occitanie, France |
| Valarie McLin | Valarie McLin | Swiss Pediatric Liver Center, Geneva, Switzerland |
| Neil Halliday | Aileen Marshall | Royal Free NHS Foundation Trust, London, UK |
| Neil Halliday | Jennifer Ryan | Royal Free NHS Foundation Trust, London, UK |
| Nikolaos K. Gatselis | George N. Dalekos | University Hospital of Larissa, Greece |
| Nikolaos Machairas | Georgios C. Sotiropoulos | Evgenideio Hospital, Athens, Greece |
| Simona Parisse | Simona Parisse | Sapienza Gastroenterology Unit, Rome, Italy |
| Sonia Blanco Sampascual | Sonia Blanco Sampascual | Hospital Universitario Basurto, Bizkaia, Spain |
| Stella Gabeta | George N. Dalekos | National Expertise Center of Greece in Autoimmune Liver Diseases, University Hospital of Larissa, Greece |
| Steven Masson | Mhairi Donnelly | Newcastle Hospitals NHS Foundation Trust, Newcastle, UK |
| Steven Masson | Jessica Dyson | Newcastle Hospitals NHS Foundation Trust, Newcastle, UK |
| Steven Masson | Mark Hudson | Newcastle Hospitals NHS Foundation Trust, Newcastle, UK |
| Steven Masson | Steven Masson | Newcastle Hospitals NHS Foundation Trust, Newcastle, UK |
| Tamara Milovanovic | Tamara Milovanovic | Clinical Center of Serbia, Belgrade, Serbia |
| Tamè Mariarosa | Tamè Mariarosa | Policlinico S.Orsola di Bologna, Bologna, Italy |
| Valérie McLin | Valérie McLin | Swiss Pediatric Liver Center, Geneva, Switzerland |
| William R Hutson | William R Hutson | West Virginia University, Morgantown, West Virginia, USA |
| Winni Aprillia Putri | Hengki Wijaya | Iskak General Hospital Tulungagung, Jawa Timur, Indonesia |
| Yasemin Balaban | Yasemin Balaban | Hacettepe University, Ankara, Turkey |
